# GENERATOR Breast DataMart—The Novel Breast Cancer Data Discovery System for Research and Monitoring: Preliminary Results and Future Perspectives

**DOI:** 10.3390/jpm11020065

**Published:** 2021-01-22

**Authors:** Fabio Marazzi, Luca Tagliaferri, Valeria Masiello, Francesca Moschella, Giuseppe Ferdinando Colloca, Barbara Corvari, Alejandro Martin Sanchez, Nikola Dino Capocchiano, Roberta Pastorino, Chiara Iacomini, Jacopo Lenkowicz, Carlotta Masciocchi, Stefano Patarnello, Gianluca Franceschini, Maria Antonietta Gambacorta, Riccardo Masetti, Vincenzo Valentini

**Affiliations:** 1 Dipartimento di Diagnostica per Immagini, Radioterapia Oncologica ed Ematologia, UOC di Radioterapia Oncologica, Fondazione Policlinico Universitario “A. Gemelli” IRCCS, 00186 Rome, Italy; fabio.marazzi@policlinicogemelli.it (F.M.); luca.tagliaferri@policlinicogemelli.it (L.T.); giuseppeferdinando.colloca@policlinicogemelli.it (G.F.C.); barbara.corvari@policlinicogemelli.it (B.C.); mariaantonietta.gambacorta@policlinicogemelli.it (M.A.G.); vincenzo.valentini@policlinicogemelli.it (V.V.); 2 Dipartimento di Scienze della Salute della Donna e del Bambino e di Sanità Pubblica, UOC di Chirurgia Senologica, Fondazione Policlinico Universitario “A. Gemelli” IRCCS, 00186 Roma, Italy; francesca.moschella@policlinicogemelli.it (F.M.); martin.sanchez@policlinicogemelli.it (A.M.S.); gianluca.franceschini@policlinicogemelli.it (G.F.); riccardo.masetti@policlinicogemelli.it (R.M.); 3 Istituto di Radiologia, Università Cattolica del Sacro Cuore, 00186 Rome, Italy; nikoladino.capocchiano@unicatt.it (N.D.C.); jacopo.lenkowicz@gmail.com (J.L.); 4 Fondazione Policlinico Universitario “A. Gemelli” IRCCS, 00186 Roma, Italy; roberta.pastorino@policlinicogemelli.it (R.P.); chiara.iacomini@guest.policlinicogemelli.it (C.I.); carlotta.masciocchi@guest.policlinicogemelli.it (C.M.); stefano.patarnello@guest.policlinicogemelli.it (S.P.); 5 Istituto di Semeiotica Chirurgica, Università Cattolica del Sacro Cuore, 00186 Rome, Italy

**Keywords:** breast cancer, DataMart, real world data, predictive model, healthcare

## Abstract

Background: Artificial Intelligence (AI) is increasingly used for process management in daily life. In the medical field AI is becoming part of computerized systems to manage information and encourage the generation of evidence. Here we present the development of the application of AI to IT systems present in the hospital, for the creation of a DataMart for the management of clinical and research processes in the field of breast cancer. Materials and methods: A multidisciplinary team of radiation oncologists, epidemiologists, medical oncologists, breast surgeons, data scientists, and data management experts worked together to identify relevant data and sources located inside the hospital system. Combinations of open-source data science packages and industry solutions were used to design the target framework. To validate the DataMart directly on real-life cases, the working team defined tumoral pathology and clinical purposes of proof of concepts (PoCs). Results: Data were classified into “Not organized, not ‘ontologized’ data”, “Organized, not ‘ontologized’ data”, and “Organized and ‘ontologized’ data”. Archives of real-world data (RWD) identified were platform based on ontology, hospital data warehouse, PDF documents, and electronic reports. Data extraction was performed by direct connection with structured data or text-mining technology. Two PoCs were performed, by which waiting time interval for radiotherapy and performance index of breast unit were tested and resulted available. Conclusions: GENERATOR Breast DataMart was created for supporting breast cancer pathways of care. An AI-based process automatically extracts data from different sources and uses them for generating trend studies and clinical evidence. Further studies and more proof of concepts are needed to exploit all the potentials of this system.

## 1. Background

In the last few years, breast cancer (BC) curability has been highly improved thanks to implementation of treatments and technologies [1]. In oncology, clinical research is usually supported through prospective clinical trials in which collected records are usually codified by an ontological system [2] and electronic case report forms (CRFs) [3]. However, prospective clinical trials require a considerable number of resources and time to obtain statistically meaningful outcomes. In addition, the results obtained after 5–10 years from a clinical trial often cannot reflect up-to-date technical needs and can be overtaken by new therapeutic choices [4]. Alongside the high-quality data from clinical trials, low-quality but high-quantity data generated by clinical practice are often not used because they are difficult to collect and analyze [5]. Real-world data (RWD) studies represent a possibility for obtaining evidence from clinical practice, because they are considered to be more representative of the patients and trends that are currently being treated. RWD are stored and potentially available inside hospital informatic systems, both in structured and unstructured formats, and carry truly relevant information applicable for different scopes (research, monitoring, alert, etc.).

The use of automated data discovery and Artificial Intelligence (AI) in medical research has also increased exponentially in recent years, and the continuous development of improved computer science and machine learning tools helps raise the efficiency of research by automating various processes that are usually either performed manually or in a suboptimal way [6]. Additionally, even more thriving applications of data discovery and AI in oncological sciences have led to the development of systems capable of substantially improving diagnostic and therapeutic choices [6,7,8]. Thanks to the automated process of AI, RWD extraction and analysis could become even more feasible without manual work, but, even more, the system could learn from RWD to predict trends and improve processes [9]. 

The primary aim of this work is to show an integrated, highly replicable approach where the use of modern technologies (e.g., data discovery, transformation, and AI-based technologies) is leveraged in order to extract, validate, and organize RWD data. This approach is applied to the domain of breast cancer and will allow doctors to organize information for patients’ treatment history in a time-effective manner, by centralizing such data from different archives distributed in the hospital healthcare systems into a single standardized repository for breast cancer real-world data (called Breast DataMart). This procedure will, in turn, enable focused studies to be much more effective in their aims. In this work, we also highlight how this DataMart can be exploited through machine learning, to obtain models for outcome prediction and development of guardian systems set up to monitor the clinical flow of patients (pts) and provide supporting info for corrective actions, e.g., in time-sensitive treatment schedules. We describe the architectural structure of the Breast DataMart, and two proof-of-concept designs intended to show the potential of the guardian systems and the automated data-extraction procedures.

## 2. Materials and Methods

### 2.1. Domain-Specific Ontology

At the very start of the project, we focused on the definition of a terminology dictionary aligned to the requirements of the clinician team, in terms of completeness of patients’ data, accuracy in the description of clinical workflow, and relationships among entities. This phase of the ontology definition was developed jointly among clinical experts and data scientists, to make sure that the mapping into the target IT framework was accurate and viable. 

### 2.2. Multidisciplinary Team and Rapid Requirement Definition

The goal of building a framework that can be extensively leveraged across multiple studies and trials has naturally led to organize a team which could offer a comprehensive view of the needs from a clinical research side, tightly connected with the technology experts for the technical design and architectural builds. A multidisciplinary team of experts was formed by radiation oncologists, epidemiologists, medical oncologists, and breast surgeons, to approach breast cancer RWD from a clinical perspective. To develop a system able to collect, transform, and organize data from different archives within the hospital IT system, data scientists and data management experts worked to capture requirements from clinical teams and translate them into fast prototypes and implementation. Combinations of open-source data science packages and industry solutions (SAS^®^ Viya framework) were used to design the target framework. To validate the DataMart directly on real-life cases, the working team defined tumoral pathology and clinical purposes of proof of concepts (PoCs). The PoCs were immensely helpful for a user-oriented approach to select, classify, and organize data in the DataMart. From the project-management perspective, the working group adopted a rapid development strategy, where the clinical team (i.e., end users) and technology staff were highly integrated in designing, prototyping, and validating intermediate and final outcomes. 

### 2.3. Breast Cancer DataMart Architecture

The working team chose breast cancer as the initial pathology to be investigated for the DataMart creation process. Besides the expertise of the working team, breast cancer was properly chosen for its high range of possible variables and different archives with relevant information in the hospital IT infrastructure. The approach to extract, transform, and organize information in the target DataMart is based on a multilayer approach: The first layer is based on the hospital IT platform, in which the retrospective data are centralized and structured in accordance with the ontology defined, and prospective data items are collected daily from physicians, analysis laboratories, and electromedical devices, to then be stored and protected with the strictest physical and logical security criteria. The working team also classified sources or “Channel Doors” from which to import data. The second layer is the DataMart structured dataset; the interchange between the first and second layer is handled with a set of IT tools: automated procedures to feed a real-time flow of data stemming from the daily clinical practice; connectors to electromedical devices (e.g., to extract radiomic data); text-mining techniques, which transform unstructured text (e.g., consultancies, exam reports, and diagnoses) into clinically relevant structured data. Raw data from production repositories are extracted in pseudo-anonymized form, to protect patients’ privacy. The third layer is the discovery one, where analytics, machine learning, and AI methods are applied to perform the studies (in our case, the PoC). The output of this semi-automated AI layer can be represented in formats which are relevant for the clinical staff through the development of easy-to-use graphical user interfaces or different forms based on the specific study (production of synthetic data, out-come-related risk scoring, etc.).

**Figure 1:**
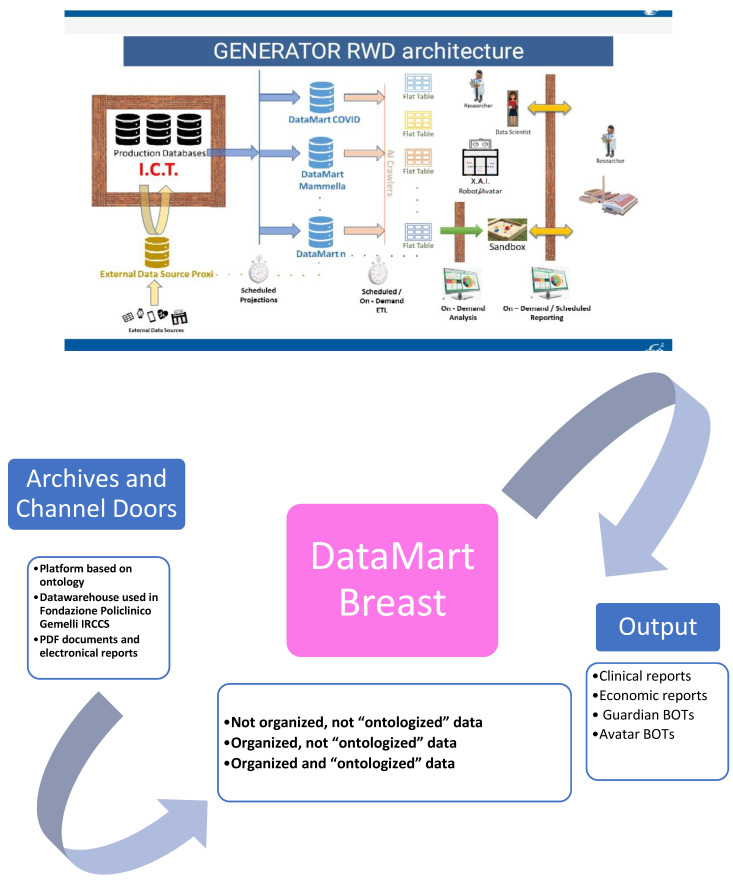
GENERATOR Breast DataMart architecture. In this figure, architecture of GENERATOR Breast DataMart is described. On the left, the sources are reported (a description is provided in Table 1). Thanks to Artificial Intelligence (AI) automatism, connection, and procedures, it is feasible to extract these data sources and deposit them inside Breast DataMart. An external server support Breast DataMart. Data extracted are available for further elaboration, such as creation of robots (or BOTs) for implementation of clinical research.

With this approach, the Breast Cancer DataMart was defined and structured as the shared global archive of all available breast cancer data inside the hospital IT system of Fondazione Policlinico Gemelli IRCCS, which will be continuously updated through the scheduled procedures (Figure 1).

Finally, to program the DataMart implementation, the working group (WG) divided future processes into 3 distinct phases:

Phase 1: proof of concept;

Phase 2: internal consent with multidisciplinary contribution;

Phase 3: dynamic DataMart with data access for monitored and authorized internal and external requests.

To verify the usability and effectiveness of the DataMart and the overall framework, the team proposed two proofs of concept (PoCs) for testing purposes. The first one was identified as a “waiting time” calculation test from surgery to radiotherapy beginning. The second PoC was set up to calculate and test a series of key performance indicators (KPIs) based on diagnostic and therapeutic performance markers. Each end-product of the two PoCs was defined as a robot (BOT) in accordance with the AI-related features, in terms of AI data governance, automated procedures, and end-user output. 

For each PoC, the definition of specific methodology and development pathways were required: DataMart access and usage (including variables selection and definition, archives and channel doors identifications, and data extraction processes), modeling phase (BOT construction), and end-user testing (BOT clinical validation).

## 3. Results

Starting from October 2019, the working team organized meetings on a regular basis, in order to keep the workflow initially defined. Meetings were both live and online. All the tasks planned for phase one of the DataMart development were completed.

Data definition: The WG defined data on the basic of their availability. Results are resumed in Table 1.

Archives and channel doors definition: Based on data definition, Multidisciplinary Team then defined where to find data for filling Breast Cancer DataMart and for capture them different “channel door” were identified. “Channel door” identified are reported in Table 2.

Waiting Time Bot: As already mentioned, this PoC addresses the waiting-time calculation from surgery to the start of radiotherapy. We believe this is a relevant testbed for two purposes: as a process BOT, to identify areas to improve and accelerate patients’ clinical paths; and as a supporting platform for interventional studies, to track the evolution of the selected cohorts. The team identified pathways for data extraction and elaboration.

ICD9 codes for diagnosis and surgery were selected for identifying pts with breast cancer who underwent surgery. We evaluated 10 main variables to extract by the text-mining technology the time lapses in which the RT was performed: Seven were structured variable, such as the date of birth or the kind of surgery, and three were unstructured variables (multidisciplinary board indication to chemotherapy, radiotherapy, or both). Text mining selected patients with multidisciplinary indication to adjuvant chemotherapy and radiotherapy. Waiting-time interval was calculated as the interval between surgery and the first day of treatment.

From January 2017 till December 2019, a cohort of 2074 patients underwent surgery for breast cancer. Between them, 655 pts were addressed to adjuvant RT alone, 113 to adjuvant chemotherapy alone, and 153 to both. Of this cohort, 1023 underwent RT in our hospital. Mean waiting time was 119 days (31–345). They were divided into three groups, based on waiting-time interval: 154 patients underwent RT within 60 days from the surgery; 407 patients, starting from 60 days after the index breast surgery and up to 90 days; and 462 patients who were treated after 90 days from surgery. Patients who came from other regions, and so, far from our center, experienced a wider delay in the beginning of RT.

**Figure 2:**
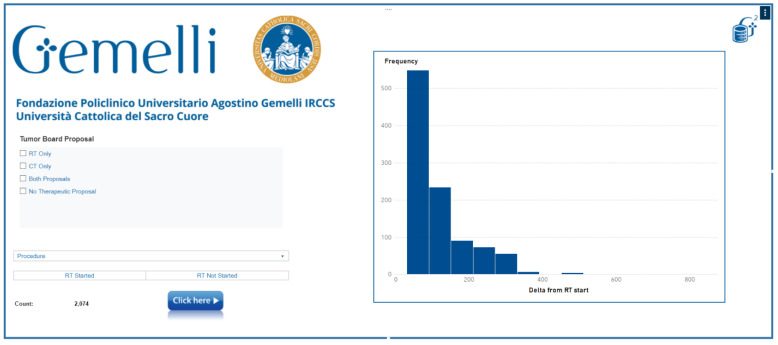
Waiting Time BOT platform.

The Wating Time BOT showed that it is feasible to extract data from different data sources inside the hospital system, to obtain an output for monitoring real-time pts’ waiting time for radiotherapy treatments (Figure 2). Output of this evaluation needs to be implemented and integrated inside the hospital system, to have an alert for managing patients’ waiting-time delay. Specific further prospective studies are needed to highlight predictive factors that can influence the timing of RT.

*KPIs Bot*: The goal of this PoC is to create, through data clustering, a group of Key Performance Indicators (KPIs) based on diagnostic and therapeutic performance [11]. Among other potential exploitations, this is a simplified example of how the DataMart can be used for rule-based patients’ recruitment. ICD9 codes for diagnosis and surgery were chosen for selecting pts with breast cancer who underwent surgery. In accordance with the aim of the study, we selected nine KPIs to be extracted (Table 3). For each KPI, variables for its definition were selected and divided in structured and not structured. The last one was extracted by text mining. Artificial Intelligence automated pathway of extraction identified 2144 patients. Five different data sources were used for data extraction.

Nine structured (age, ICD9 diagnosis, ICD9 surgery, ICD9 diagnostic exams, data of beginning chemotherapy and/or radiotherapy, data of recovery and dismissal, and data of pathology exam) and four not-structured variables by text-mining elaboration (subtypes, staging, and multidisciplinary board therapeutic indications) for KPIs’ calculation were identified. Extraction populated all KPIs, and mean rate of data extraction in text-mining elaboration was 78% and 88.3%, respectively, for staging and subtypes’ characterization. KPIs’ performance was, respectively, (1) 20.91%, (2) 17.88%, (3) 26.9%, (4) 0.25%, (5) 1.72%, (6) 44.6%, (7) 92.2%, (8) 95%, and (9) 67.3%.

KPIs’ extraction was feasible, even if further validation is necessary to implement data extraction and optimize quality of data, to create a simultaneous evaluation of them, integrated inside the hospital system.

## 4. Discussion

Artificial Intelligence (AI) is the ability of technology applications to accomplish any cognitive task, at least as humans [12]. Even more than ever, AI is transforming our lives and job-automating processes, and it is becoming an indispensable tool for research and development of technology. Creation of a system based on AI requires the use of neural network replications that are capable of answering some questions, identifying specific patterns of data, and learning from them. For this, it is fundamental to create algorithm connections on which system AI technologies need to run. It has been already reported by Carter et al. that breast cancer care was always supported by AI applications since the 1970s, and now it is even more integrated in diagnostic systems [13], for example, in mammography implementation [12,14]. There are many single experiences of AI application in breast cancer care. An example of this issue is reported by Schaffter et al., in a study in which AI was applied to build algorithms for interpretation of screening mammography [15]. Another study by Pantanowitz L et al. reported application of AI to pathology activity of quantifying mitotic figures in digital images of invasive breast carcinoma with implementation of accuracy and overall time savings, respectively, in 87.5% and in 27.8% of cases [16]. 

Moreover, it is demonstrated that, thanks to AI application, it is possible to save time, lower costs, and raise efficacy [12]. 

In our experience, we created an AI connection to allow not only storage of all data repository of breast cancer patients who were treated in our hospital, but also a system that is capable of being interrogated for different purposes. In fact, data stored in our Breast DataMart were analyzed and used to create two systems: Waiting Time BOT for monitoring waiting time from surgery to radio-therapy, and KPIs’ BOT for evaluating different aspects of breast unit performance. In both cases, BOT were comparable with clinical practice and the literature. In fact, waiting time in breast cancer represents a key point of treatments, and its delay can lead to reduced efficacy in terms of breast cancer outcomes [17,18,19,20]. In particular, a waiting time of 12 weeks or more from surgery to the start of radiation (for patients who are not candidate to adjuvant chemotherapy) and a waiting time of six weeks or more from completion of chemotherapy to start of radiation (for patients who are candidate to adjuvant chemotherapy) are associated with worse event-free survival after a median follow-up of seven years [17]. Given that radiotherapy should be started as soon as reasonably possible, a monitoring system such as Waiting Time BOT could allow not only to track possible delays in pts’ pathway of care, but also to learn to predict factors that can be associated to this delay and can be prevented. On the other hand, the KPIs BOT, which allows users to track the performance of breast tumor pathway of care, is based on a system of indicators published by Altini et al. [11] in 2019. In this system, multidisciplinary evaluation is fundamental, but in the hospital system, services provided by the various departments can be reported on different informatic platforms or archives. This usually requires a data entry or data manager to report the folder manually inside CRFs for data collection [21]. In the literature, systems for tracking breast unit performance are reported, for example, EUSOMA, which is used for quality assurance [22]. However, the GENERATOR Breast DataMart does not want to replace these already established systems, but rather offers the possibility to search for data sources automatically for any type of analysis and can therefore be integrated with them.

Beyond the individual project with AI application, there is a multitude of data that could be analyzed by different prospective for implement patterns of care by the following:

- GUARDIAN ROBOT: an instrument that is able to alert the physician on determined items, capable to learn by data implementation.
- PREDICTIVE ROBOT: an instrument capable to predict trend of outcomes capable to learn by data implementation.
- DESCRIPTIVE ROBOT: an instrument capable to describe determined trends that can be used for cost/effectiveness purposes.
- AUTHOMATED ROBOT: an instrument that is linked to some diagnostic and therapeutic procedure, to reduce time of elaboration and lead physicians to more precise results.

Breast DataMart is a dynamic system based on AI, with the purpose of connecting data patterns from different sources, answer specific questions, and learn from data analyses, to implement outputs. The PoCs we performed in this study demonstrate that it is feasible to achieve this purpose for breast cancer care, using simple pathways. We interrogate the system about waiting-time data, the system returns data of interest, and it learns from them, constructing a “guardian system” to predict waiting time of patients and surgery data. On the other side, we created a second system of data elaboration by KPIs analysis. DataMart system was trained to find and return data of interest for analysis. Final elaboration allows clinicians to have a system integrated in the hospital system for on-line contemporary analysis. DataMart goals are not only to obtain a single PoC, but to have an entire data repository for breast cancer continually analyzed and processed, with the possibility to perform unlimited queries. Results of these queries can be integrated in the Hospital System for Guardian or Avatar robot system. Future application of Breast DataMart system in breast cancer care is addressed to reduce biases in patterns of care, manage heterogeneity of disease, and create algorithms for implement cost/effectiveness. 

Standardized Data Collection (SDC) is a recent methodology for extract and use of real-world data. It is based on the concept that, besides structured data available by clinical trials, we have a multitude of low-quality big data inside electronic and paper folders, from which evidences can be generated, also closer to clinical practice [23,24,25,26]. AI introduction leads to the evolution concept that modern oncology not necessarily needs to be built only on SDC, but here AI application allows us to use real-world data to obtain data classificatory, predictive model, or guardian for clinical practice also in a not-time-consuming process. In this way, a system such as Breast DataMart, which we developed, becomes a dynamic application of SDC-captured data, with automated possibility of on-line queries. Moreover, DataMart AI technology, thanks to neural networks applied to building unstructured data and retrieving data through text mining, ensures that otherwise lost data are included in its system. In fact, it is possible to also recover from the hospital system PDF documents and electronic reports. The guarantee that the data contained in them are certified is linked to the officiality of these reports. Finally, since the DataMart is linked to the hospital system, its outputs can be integrated, in turn, into clinical practice, as alert systems, to obtain predictions or simply to describe useful trends to manage cost/effectiveness items.

## 5. Conclusions

GENERATOR Breast DataMart was created for supporting breast cancer pathways of care. An AI-based process automatically extracts data from different sources and uses them for generating trend studies and clinical evidence. For testing its use, two proof of concepts on waiting time and KPIs’ calculations were built and validated. Further steps will include DataMart population with all data online in the hospital system and to start queries to implement clinical practice. Further studies and more proof of concepts are needed to exploit all the potentials of this system.

## Figures and Tables

**Table 1:** Data definition and classification, according to their availability.

Data Definition and Classification		
Definition	Description	Example
**Not organized, not “ontologized” data**	Data to be constructed from other records and not captured by a pre-existing ontology system	For example, “Therapeutic indications from a Tumor Board”
**Organized, not “ontologized” data**	Records constructed but not captured by a pre-existing ontology system from begin	For example, “Data of radiotherapy beginning” or ICD9 code for diagnosis
**Organized and “ontologized” data**	Data captured by a pre-existing ontology system that can be directly recovered or is deposited in another software system	For example, data collected by data manager and data entry on dedicated web or hub systems

**Table 2:** Archives and channels doors definitions.

Archives and Channels Doors Definitions			
**Definition**	**Description**	**Type of Data Extraction**	**AI Technologies and Automatisms Performed**
**Platform based on ontology**	Platform in use in our hospital for standardized data collection (BLADE, RedCAP, etc.). In this platform it is integrated a shared ontology that codifies data in unique, non-ambiguous way.	Organized and “ontologized” data	NEURAL NETWORKS
**Datawarehouse used in Fondazione Policlinico Gemelli IRCCS**	Data warehouses in use in our hospital for clinical assistance (SI, Aria, Speed (advanced evolution of Spider [10], Armonia, TrackCare, etc.). In these systems, data are codified based on clinical practice (e.g., Hb value, date of surgery, etc.), and are data validated by conventional clinical use	Organized, not “ontologized” data	NEURAL NETWORKS
**Text mining extraction from PDF documents or electronic reports**	All the electronic documents present in previous archives in which a procedure of text-mining extraction was applied to recover non-structured data. This is a very relevant part of data extraction, because we can recover a big quantity of granular information and translate it into structured data for usage in clinical practice and research.	Not organized, not “ontologized” data	TEXT MINING AUTOLEARN NEURAL NETWORKS

**Table 3:** KPIs description.

KPI Name	KPI Description
**KPI pre-surgery**	percentage of stage I and II breast cancer patients who underwent at least one radiological exam in the 60 days prior to the breast surgery
**KPI post-surgery**	percentage of stage I and II breast cancer patients who underwent at least one radiological exam within the 60 days after the surgery
**KPI follow-up **	percentage of stage I and II breast cancer patients who underwent at least one radiological exam from 60 days after the index breast surgery and up to 365 days after this surgery
**KPI Subsequent Breast Reconstruction/Axillary dissection**	percentage of patients with BC who underwent subsequent surgery
**KPI subsequent breast surgery**	percentage of patients with BC who underwent subsequent surgery following a partial resection
**KPI chemotherapy timing **	percentage of patients with BC who, as candidates for chemotherapy, initiated adjuvant treatment within 60 days of the index breast surgery
**KPI radiotherapy timing**	Percentage of patients who initiated radiotherapy within 180 days of the last surgery
**KPI time of recovery**	Percentage of patients who presented a recovery time in less than 7 days
**KPI pathology exam**	Percentage of patients who received a pathology exam in less than 15 days

KPI, Key Performance Indicator.

## Data Availability

The data presented in this study are available on request from the corresponding author.
